# Direct Acrylation of Soybean Oil and the Influence of the Acrylation Degree on Waterborne Acrylic Systems

**DOI:** 10.3390/polym16162355

**Published:** 2024-08-20

**Authors:** Beatriz Perez, Noelia Blanco, Haizea Villaverde, Oihane Echeverria, Olga Gomez de Miranda, Raquel Rodriguez

**Affiliations:** TECNALIA, Basque Research and Technology Alliance (BRTA), Paseo Mikeletegi 2, 20009 Donostia-San Sebastian, Spain

**Keywords:** acrylated soybean oil, direct acrylation, miniemulsion polymerization, copolymerization, hydrophobicity, oleophobicity

## Abstract

The direct acrylation of soybean oil was investigated by the activation of soybean oil’s (SO’s) internal fatty unsaturation with acidic catalysts. The effect of the catalyst and the reactant ratio with respect to the unsaturation and reaction time on the direct acrylation process were explored. ASO (acrylated soybean oil) with acrylation degrees (the number of acrylate molecules introduced in a triglyceride molecule) between 1.6 and 2.55 were obtained. The effect of the ASO acrylation degree on copolymerization processes was investigated. The resulting monomers were successfully copolymerized with meth(acrylate) monomers by the miniemulsion polymerization process, favoring the droplet nucleation mechanism and showing conversions higher than 97%. The acrylic–ASO copolymers presented lower Tg and higher hydrophobicity and oleophobicity than the acrylic copolymer.

## 1. Introduction

Over the last few years, the polymer industry has moved to more environmentally friendly processes due to more restricted environmental regulations and public concern. In this sense, waterborne processes appear as one of the most widespread green strategies. However, this strategy mainly depends on fossil-based raw materials. During the last few years, great effort has been put into searching for alternatives based on renewable resource monomers to produce novel polymers that are able to substitute their petroleum-based counterparts. The monomers for this purpose come from different forms of biomass feedstock, such as vegetable oils and lipids, terpenes, lignin derivatives, carbohydrates and proteins [1].

Vegetable oils have become the most important renewable feedstock in the replacement of petroleum-based sources, particularly in the production of biobased polymers [2,3] in the chemical industry due to their abundance, biodegradability, low cost, nontoxicity, and ability to undergo versatile organic reactions. They consist predominantly of esters of glycerol with three fatty long-chain acids (triglycerides) with varying compositions of fatty acids [4,5,6,7,8]. Different reactions, including hydrolysis, transesterification, the amidation of ester groups, epoxidation, hydrogenation, and metathesis for double bonds, are considered for the synthesis of sustainable biobased monomers.

In considering the use of vegetable oil as a polymer precursor in free-radical polymerizations, the double bonds in vegetable oils can be functionalized through an epoxidation reaction following an acrylation reaction by incorporating an acrylic group in the triacylglycerol structure. This epoxidation reaction consists of internal alkenes of vegetable oils that react using oxidizing systems to form epoxy groups. Four epoxidation methods can be found in the literature: an in situ method with peracids in the presence of an inorganic soluble or supported catalyst; organic or inorganic peroxides, using transition metals or enzymatic species as catalysts; via halohydrins formation; or using molecular oxygen [9,10,11] which are known as epoxidized acrylic oils [12,13,14,15,16].

Soybean oil is a particularly interesting resource because it is abundant and cheap. Several studies have been conducted showing the possibilities that this raw material presents in free radical polymerization processes. Saithai et al. [17] investigated the effect of the epoxidation process route on the synthesis of acrylated epoxidized soybean oil/methyl methacrylate copolymer by the bulk polymerization process. It was found that the epoxidation process had an influence on the polymer properties, such as thermal stability and glass transition temperature (Tg). Moreover, they observed that as the percentage of the acrylate group increased, Tg increased and the thermal stability improved. Due to the low water solubility of these macromonomers, their incorporation into the conventional emulsion polymerization process is a challenge. Long aliphatic chains from fatty acids have difficulties when diffusing through the aqueous phase from the monomer droplets to the polymer particles [18]. In this sense, the miniemulsion polymerization process has emerged as a promising alternative because monomer droplets are the main loci of particle formation [19,20].

Several authors have used miniemulsion polymerization to (co)polymerize vegetable oil-derived macromonomers with meth(acrylite) monomers. Moreno et al. [21] found that the miniemulsion polymerization process was the proper process for synthesized linolenic acid-based latexes. They found that the presence of labile allylic hydrogens undergoing chain transfer reactions during the polymerization process controls the kinetics and properties of the systems.

Quintero et al. studied the copolymerization of maleinized vegetable oil [22] using miniemulsion polymerization [23]. The maximum amount of vegetable oil incorporated on the copolymer was 35 wt%, and it was found that unsaturated bounds preserved during the polymerization process allowed an oxidative cure during the drying process.

Bunker et al. [24] conducted a comparative study of the copolymerization of acrylated methyl oleate with methyl methacrylate, 1,4-butanediol diacrylate, and 2-ethylhexyl acrylate using the emulsion and miniemulsion polymerization processes. It was observed that the reaction time decreased from 18 h to 1 h and that the surfactant concentration could be reduced from 15 wt% to 2 wt% when using miniemulsion polymerization. Furthermore, copolymers with suitable properties for application as pressure-sensitive adhesives (PSAs) were obtained.

The copolymerization of styrene with transesterified soybean oil, acrylated through the double bond of aliphatic chains, was also performed via miniemulsion polymerization [25]. In this system, increasing the concentration of acrylated fatty acid methyl ester (AFAME) from soybean oil resulted in an increase in the latex particle size and a decrease in the total monomer conversion. This was explained by the increase in viscosity at an AFAME/styrene molar ratio higher than 5/95. Moreover, both the glass transition temperature and the thermal stability decreased, whereas the gel fraction increased when the proportion of the AFAME increased. Ricinoleic acid (castor oil) was also acrylated through the internal double bonds by Laurentino et al. [26], then copolymerized with MMA by miniemulsion polymerization to prepare a variety of polymers with Tg ranging from 50 to 124 °C. Similar drawbacks (bigger particle size, lower polymerization rates, and lower molar masses) were encountered as the acrylated ricinoleic acid concentration increased.

Other authors have modified the structure of the fatty vegetable oil, achieving acrylated epoxidized methyl oleate, the structure of which allows mass transfer during the emulsion polymerization process. In this sense, Jensen et al. [27] synthesized copolymers of styrene and acrylated epoxidized methyl oleate. They found that the glass transition temperature and the average molar mass of the polymer decreased as the concentration of the comonomer derived from vegetable oil increased in the polymerization medium.

**Figure 1:**
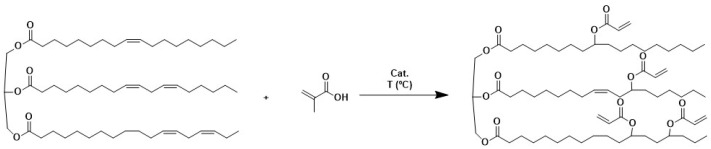
Partial acrylation of SO with AA in one-step route.

Although AESO seems to be an environmentally friendly material, the epoxidation process should be improved using more green chemistry [28]. In this sense, a direct acrylation route appears as a greener alternative to obtaining acrylated vegetable oils, avoiding intermediate epoxidation reactions (Figure 1).

The direct addition of carboxylic acids to double bonds is not as straightforward as the reaction of acid to epoxides. It was found that the acidity and the molecular size of the carboxylic acid controlled the reaction; for example, acrylic acid (AA), which presents a moderate acidity (pKa 4.25) and a small molecular size (three carbons), favors the reaction [29]. Moreover, Adnan et al. [30] found that the intermolecular and intramolecular additions of alkenes to double bonds require more extreme operational conditions than the ring opening of epoxides principally due to the steric hindrance of the double bonds, which require co-reactants of high acidic strength. Zhang et al. [31] defined a one-step route for the direct acrylation of soybean oil and acrylic acid using Boron trifluorride etherate (BF_3_·Et_2_O) as the catalyst. They found that the reaction stoichiometry and reaction time controlled the conversion of the double bonds of SO. The number of acrylate groups could reach 3.09 per triglyceride molecule, and copolymerization with styrene using a bulk polymerization process was demonstrated. In addition, Zhang et al. [32] found that AA is prone to auto-polymerization at high temperatures, leading to side reactions.

Moreover, the use of vegetable oils can control the final properties of the polymers to contribute to the transition of a safe and circular economy [13,33]. In the case of soybean oil, it is a naturally hydrophobic oil that allows the increased use of water and chemical resistance when used in coating formulations [34].

The main objective of this study is to synthesize and characterize direct acrylated soybean oil and to study its behavior as a polymer precursor in combination with commercial acrylic monomers in the miniemulsion polymerization process. The effect of process variables on the acrylation degree of soybean oil was evaluated (catalyst and AA molar ratio to soybean oil double bonds and reaction time). The influence of ASO comonomers on the miniemulsion process behavior and final properties of the ASO copolymers and conventional meth(acrylate) copolymer were discussed (glass transition temperature and hydrophobicity/oleophobicity).

## 2. Materials and Methods

### 2.1. Materials

Soybean oil was supplied by Mysticmomentsuk.com, anhydrous AA (with a purity of 99%), was acquired by Fluka, and hydroquinone (99.5%) was purchased from Acros Organics (BV-Geel, Belgium). The catalyst BF_3_·Et_2_O (48% in diethyl ether) was purchased from Sigma-Aldrich. Finally, ethyl acetate with a purity of 99.8% was acquired at Fisher Scientific, NaCl (99.5%) was obtained from Fluka (Buchs, Switzerland) and Na_2_SO_4_ anhydrous (99%) was obtained from Sigma-Aldrich (Madrid, Spain). Technical-grade monomers, methyl methacrylate (MMA, purity 99%), butyl acrylate (BA, with 99% purity), and acrylic acid (AA, with 99% purity) supplied by Aldrich, were used without purification. Stearyl acrylate (SA, Sigma-Aldrich) was used as a costabilizer, and SDS (sodium dodecyl sulphate, Sigma-Aldrich) was used as a surfactant. Ammonium persulfate (APS, Panreac, Barcelona (Spain)) was used as the initiator. Distilled water was used throughout the work.

### 2.2. Synthesis of Acrylated Monomers (ASO) in a One-Step Process

SO was mixed with AA, hydroquinone, and with BF_3·_EtO_2_ as a strongly acidic catalyst in a glass reactor with a condenser and reacted under magnetic stirring. The mixture was allowed to react for a desired time at 80 °C. When the reaction finished, it was cooled down, and the excess of AA was evaporated at 45 °C under a vacuum. The resulting product was purified by diluting it with ethyl acetate and washing it several times with a brine solution to remove the unreacted AA, hydroquinone, and catalyst until the pH of the aqueous phase became neutral. The organic phase was then dried with Na_2_SO_4_, filtered, and evaporated under a vacuum. The resulting product was named the partial ASO.

### 2.3. Copolymerization of ASO and Acrylic Monomers by Miniemulsion Polymerization

Miniemulsification: The organic phase was prepared by dissolving the costabilizer in the monomer mixture, and the aqueous phase was formed by dissolving the surfactant in water. Both phases were mixed for 10 min using a magnetic stirrer at 1000 rpm. The coarse emulsion was sonified in a UIP1000hdT Ultrasonic Homogenizer at 70% for 20 min. Table 1 summarizes the formulations of the miniemulsions used. The code describing the miniemulsions is as follows: B refers to batch polymerization, and for ASO, the first number refers to the biomonomer and the second number to its concentration. Three different ASO biomonomers were selected for this study; the reaction parameter and their acrylation degree (AD) are shown in Table 2.

Polymerization processes: Batch polymerizations were carried out in a 400 mL glass reactor equipped with a reflux condenser, stirrer, sampling device, and nitrogen inlet. The reaction temperature (70 °C) was set to a constant by controlling the temperature of the fluid in the jacket by means of a thermostatic bath and a heat exchanger. The miniemulsion was added to the reactor and kept under stirring and a nitrogen atmosphere (12–15 mL/min). When the reaction temperature was reached, the aqueous initiator (APS) solution was injected.

### 2.4. Characterization Techniques

The chemical structure of the ASO monomers and acrylic–ASO copolymers was analyzed by a Bruker Alpha Platinum ATR spectrometer, which collects the spectrum at a resolution of 4 cm^−1^ in absorbance mode, at a range of 400–4000 cm^−1^. For each analysis, a total of 32 scans were carried out, respectively, and they were analyzed by OPUS 6.5 software. ^1^HNMR spectroscopy was used to estimate the acrylation degree of the monomers, which was obtained by the integration of the peak areas referenced to stable protons considered as the reference peaks and to determine the complete conversion of comonomers. A Bruker Avance 400 MHz spectrometer equipped with a QNP z gradient probe was used at room temperature. The signals obtained were represented in parts per million (ppm) in relation to the internal standard tetramethylsilane. The spin multiplicity was expressed by s = singlet, d = doublet, t = triplet, q = quartet, and m = multiplet. The analysis was made by dissolving the ASO samples into deuterated chloroform (40 mg/mL) and deuterated DMSO in the case of acrylic–ASO copolymers.

Miniemulsion droplet (dd) and latex particle (dp) z-average diameters were measured by dynamic light scattering using a Zetasizer Nano Z (Malvern Instruments Ltd., Malvern, Worcestershire, UK). Miniemulsion was diluted in distilled water (no differences were observed when distilled water or distilled water saturated with MMA was used), and the droplet size was measured within 10 min of its preparation.

Copolymer conversion was analyzed by gravimetry.

The polymer glass transition temperature (Tg) was measured by a differential scanning calorimetry (DSC) Q 100 (TA Instruments, New Castle, DE, USA). Samples were weighted into aluminium pans, and covers were sealed into place. An empty pan was used as a reference. To eliminate thermal history, two scanning cycles of heating–cooling were performed for each sample at a heating rate of 10 °C/min in the temperature range of −70 °C to 200 °C.

The obtained latexes were applied on kraft paper with a baker applicator with a 100 μm thickness. The coated papers were air-dried at room temperature for 48–72 h before conducting the analysis of water and hexadecane contact angle measurements.

## 3. Results and Discussion

### 3.1. ASO Monomer

#### 3.1.1. One-Step Process

**Figure 2:**
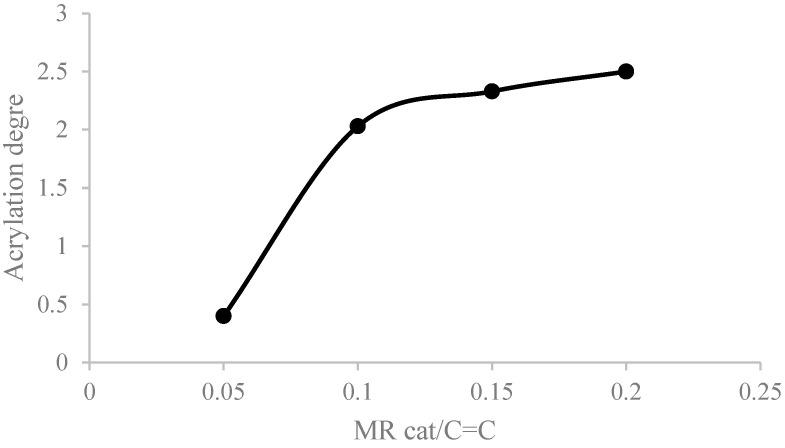
Effect of the catalyst/C=C ratio on the acrylation degree of SO.

Figure 2 shows the effect of the molar ratio between the catalyst and the internal unsaturation of the fatty chains (MR catalyst/C=C) respect to the acrylation degree. The content of double bonds in soybean oil was determined by iodine value titration [35]. On the other hand, the acrylation degree of the product (the number of acrylate molecules introduced in a triglyceride molecule) was measured by ^1^H-NMR. (Information on Supplementary Materials) This parameter was considered to evaluate the evolution of the reaction [31].

It was observed that an important restriction occurred in the reaction kinetics with lower amounts of the catalyst. An MR lower than 0.1 showed that the reaction could not be initiated. The same result was observed by Zhang et al. [32]. The authors employed high catalyst amounts to obtain relevant acrylation yields, which increased the cost of the process and made the purification steps more difficult. Higher catalyst percentages positively affect ASO acrylation, with the most convenient being an MR of 0.2. Higher values have lower effects when increasing the acrylation levels.

**Figure 3:**
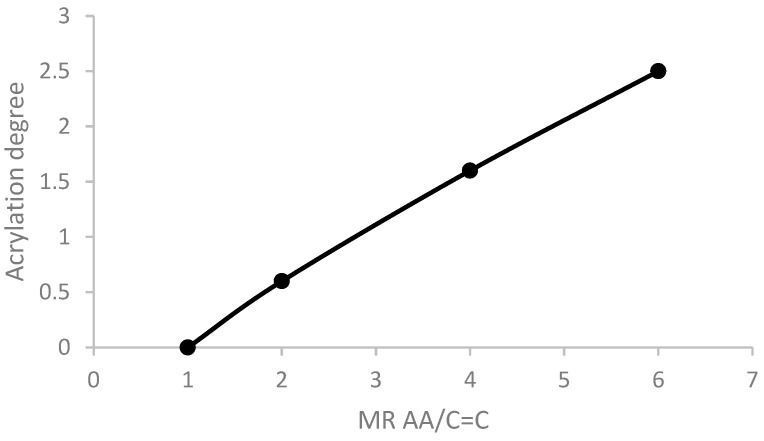
Effect of AA/C=C ratio on the acrylation degree of SO.

Figure 3 shows the effect of the molar ratio between the AA amount and the internal unsaturation of the fatty chains (MR AA/C=C) on the acrylation degree. It was found that if the catalyst MR AA/C=C was lowered from 6 to 4, the acrylation degree decreased from 2.4 to 1.6, respectively. Moreover, using ratios lower than two resulted in less than one molecule of AA being inserted per soy oil molecule. In the same way, Zhang et al. [31,32] also evidenced that an excess amount of AA had a positive impact on the acrylation degree.

**Figure 4:**
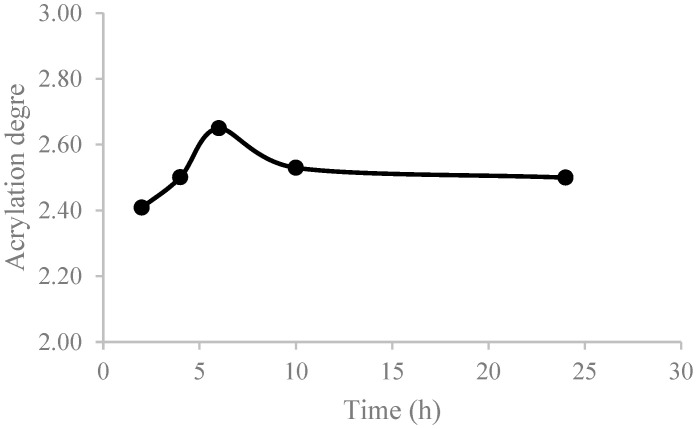
Effect of time on the acrylation degree of SO.

Figure 4 shows the influence of reaction time in the direct acrylation of vegetable oils. It can be noticed that time directly influences the acrylation of SO until 6 h (MR AA/C=C 6 and MR cat/C=C 0.2) (Figure 4). After 6 h of the reaction, the acrylation of the SO is lowered and remains constant. This could be due to undesired oligemerization reactions related to acrylic functionalities.

#### 3.1.2. Characterization

**Figure 5:**
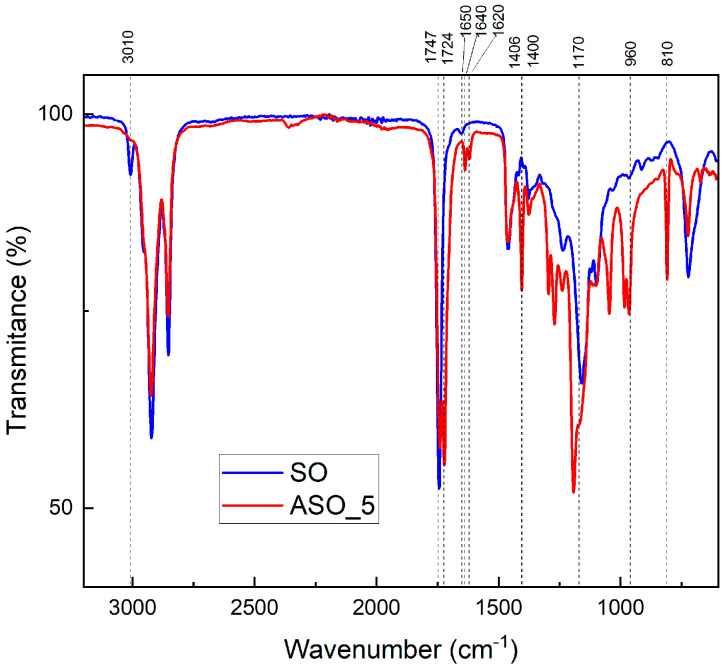
FTIR spectrum of SO (blue) and ASO (red).

The chemical structure of the synthesized ASO monomers was confirmed by FTIR. The spectra below show the acrylated monomers obtained by the direct addition of SO with AA and SO (Figure 5).

Some characteristic peaks were observed in the SO spectrum as follows: the peak at 1747 cm^−1^ refers to the carbonyl groups in TGs, and the peak at 1650 cm^−1^ is assigned to the cis C=C stretching of the vegetable oil. After the acrylation reaction in the ASO spectrum, the C–H stretching vibration of cis C=C–H of SO at 3010 cm^−1^ disappeared, and a new peak at 1724 cm^−1^ was assigned to the vibration of C=O for the acrylate groups. Moreover, the ASO spectrum presented several peaks that confirmed the acrylation of SO: the C=C vibration of the acrylate at 1640 and 1620 cm^−1^; the scissoring vibration and the rocking vibration of CH_2_ in CH_2_=C- in the acrylate group at 1400 and 960 cm^−1^; the vibration of CH in acrylate CH= at 1296 and 1272 cm^−1^; C-O-C of the acrylate groups at 1170 cm^−1^; double bonds stemming from the terminal acrylate groups at 810 and 1406 cm^−1^; and the vibration of CH_2_= in the acrylate groups at 984 and 966 cm^−1^.

### 3.2. ASO–Acrylic Copolymers

#### 3.2.1. Batch Polymerization

**Figure 6:**
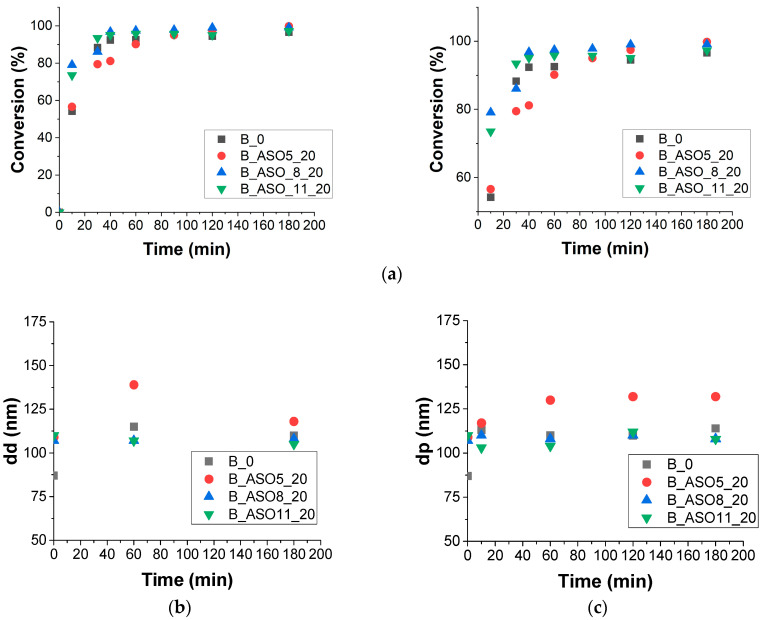
(**a**): evolution of conversion in batch miniemulsion polymerization with varying ASO biomonomers. (**b**) Effect of ASO biomonomers on droplet size (dd) and (**c**) copolymer particle sizes (dp) and their evolution along the polymerization process.

Figure 6a shows the conversion evolution for batch polymerization with ASO monomers presenting different acrylation degrees. All processes showed a fast polymerization rate. Notice that all miniemulsions presented 4 wt% of SA to avoid kinetic instabilities. Figure 6a,b present the droplet and particle size measured by light scattering along the polymerization process. The stability of the miniemulsions was controlled by the presence of the ASO monomer, and this stability was higher in the case of ASO_8 (AD) and ASO_11 (AD). Table 3 presents the number of droplets and particles calculated from the average diameters measured by light scattering. The reaction carried out without ASO (B_0) and B_ASO5_20 presented the lowest Np/Nd ratio. This behavior was reported previously [36] and attributed to poor droplet stability, and a high number of droplets was obtained with respect to the final number of particles. In the case of B_ASO8_20 and B_ASO11_20, droplets were the main loci of particle formation, and the particle size remained constant along the polymerization process. The different behaviors of the ASO monomers can be ascribed to different acrylation degrees because it can be assumed that the propagation rate through the methacrylate group is similar in all reactions. A similar behavior was observed when monomers with different alkyl chain structures were investigated [21].

These results show that droplet nucleation is strongly affected by miniemulsion stability and that ASO can act as a hydrophobe depending on its chemical structure. This effect was also found by Quintero et al. [23] using a soybean acrylate macromonomer (SAM) as a copolymerizable hydrophobe in miniemulsion polymerization.

#### 3.2.2. Characterization

**Figure 7:**
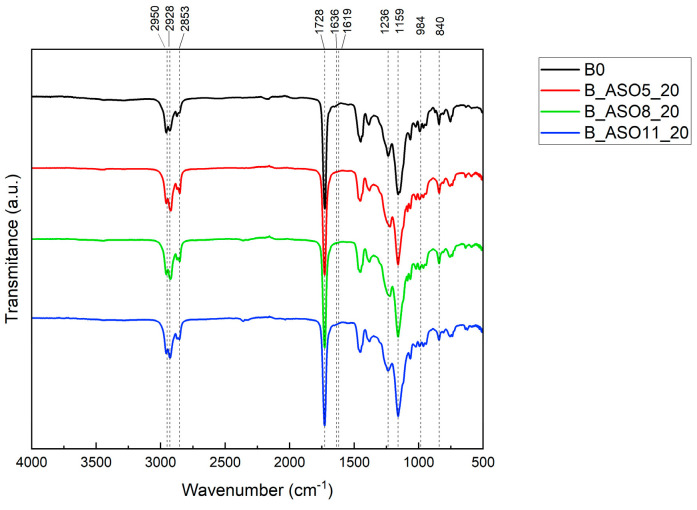
FTIR spectrum of acrylic (B_0) and ASO-acrylic copolymers (B_ASO5_20, B_ASO8_20 and B_ASO11_20.

In the case of neat copolymer B_0 and ASO acrylic copolymers (B_ASO5_20, B_ASO8_20 and B_ASO11_20), Figure 7 shows the main characteristic peaks which appeared at absorption frequencies: C-H vibrational stretching modes (2950 cm^−1^ as CH_2_, 2928 cm^−1^ s CH_3_, and 2853 cm^−1^ s CH_2_), a sharp intense carbonyl stretching vibration at 1728 cm^−1^, and two absorption bands observed at 1236 cm^−1^ and 1159 cm^−1^ attributed to the C-O-C stretching vibration. A vibrational stretch of O-CH_3_ at 1159 cm^−1^ and -CH_2_ rock at 840 cm^−1^ was also observed [23]. In these cases, no peaks were observed at 1636, 1619, and 984 cm^−1^, which represent the -CH=CH_2_ stretching of the vinyl functional group, C=C in the acrylated group and -CH_2_=CH(CO)-O- in the out-of-plane bending of vinyl functional group (Figure 5).

**Figure 8:**
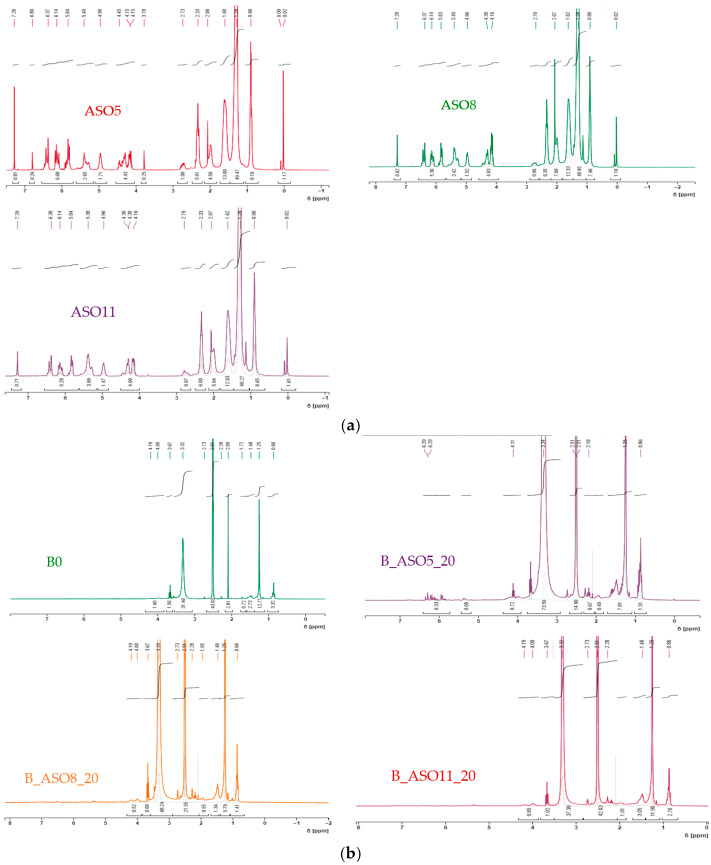
(**a**) ^1^H-NMR spectrum of ASO biomonomers in deuterated chloroform (ASO5 blue, ASO8 red and ASO11 green). (**b**) ASO–acrylic copolymer ^1^H-NMR spectrum in deuterated DMSO (B_0 black, B_ASO5_20 blue, B_ASO8_20 red, and B_ASO11_20 green).

To confirm that no unreacted ASO was presented on ASO–acrylic copolymers, ^1^H-NMR analyses were carried out. Figure 8a shows the ^1^HNMR spectra for ASO monomers. In all cases, signals from the vinylic hydrogens (5.7–6.5 ppm) and allylic hydrogens (around 4.25 ppm) were detected. Looking at Figure 8b, only the B_ASO5_20 copolymer shows peaks at 5.7–6.5 ppm, meaning that only the ASO monomer, which presented a higher acrylation degree, had an unreacted ASO monomer. This can be related to the fact that not all the acrylic functionalities of ASO5 reacted under the experimental conditions carried out.

**Figure 9:**
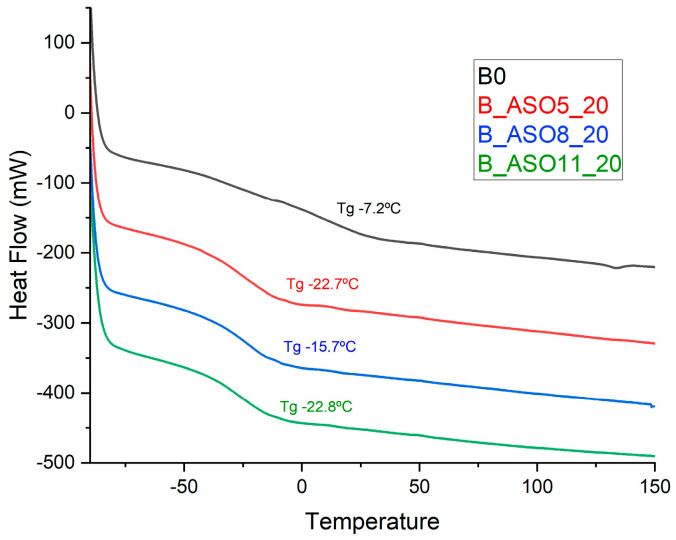
Thermal transition of ASO-acrylic copolymers and acrylic copolymer.

The analysis of Tg data measured by differential scanning calorimetry (DSC) shows that the presence of ASO in the resulting copolymers decreased the glass-transition temperature in comparison to that of the acrylic copolymer (Figure 9. This behavior was also observed by Demchuk [37]. The Tg of the samples prepared from ASO5 and ASO11 was −22.7 °C and −22.8 °C, which was lower than that of the sample from ASO8 (−15.7 °C). This result suggests that the ASO incorporation degree into the copolymers is at least one of the parameters that control copolymer Tg. Moreover, looking at Figure 9, it can be concluded that the biobased renewable monomer ASO can be used as a soft component in replacement of the traditional petroleum monomers to tune the Tg of ASO–acrylic copolymers and the percentage of ASO on the copolymer also controls the Tg value.

**Figure 10:**
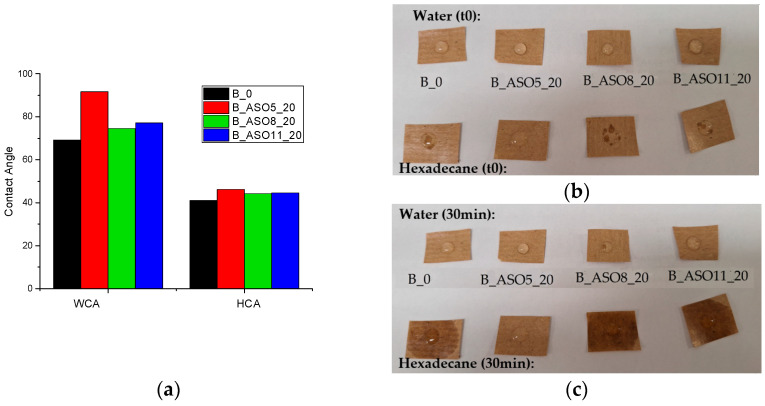
(**a**) Contact angle values WCA (water) and HCA (hexadecane) for (**b**) water and hexadecane drops at t0 and (**c**) water and hexadecane drops after 30 min.

The water and hexadecane repelling properties of the coated kraft papers were evaluated by water and hexadecane contact angle analysis (WCA and HCA) and by putting water drops on square-shaped paper, as shown in Figure 10. In terms of water repellence, coated kraft paper with an acrylic copolymer (B_0) shows higher hydrophilicity than ASO copolymers. The most hydrophobic ASO copolymer was reference B_ASO5_20, which corresponds to the latex in which not all the acrylic functionalities reacted. This could be related to the distribution of the polymeric chains into the polymer particles, where chains with free acrylic groups present more hydrophilicity than non-free acrylic groups and, thus, they can be located during the polymerization process at the shell of the B_ASO5_20 copolymer, giving the copolymer a more hydrophobic final property. On the other hand, the acrylic copolymer also presented lower oleophobicity, and the ASO copolymers presented a similar behavior. To obtain further insight into the water and oil-repelling properties, water and hexadecane drops were put on the coated papers and the shape of the drops was observed when just added (t0) and after 30 min. In the case of water, at t0, samples presented excellent water-repelling properties, but after 30 min, the B_ASO5_20 sample had some water absorption. On the other hand, all samples presented hexadecane absorption at t0 and after 30 min except for B_ASO5_20, which showed the highest water and oil contact angles.

## 4. Conclusions

Soybean oil can be acrylated by a direct acrylation route. The acrylation degree is controlled by the operational conditions. It was found that a minimum amount of catalyst is required to achieve the acrylation of soybean oil. After achieving a maximum acrylation degree after 6 h, ASO presents a reduction in the acrylation yield due to the oligomerization reactions of acrylic functionalities. It has been shown that ASO can act as an effective monomer droplet stabilizer for miniemulsion polymerization, giving a droplet size of around 110 nm. ASO–acrylic systems with 20 wt% of ASO were synthesized by miniemulsion polymerization. ASO, with the highest acrylation degree, presented unreacted acrylic functionalities on the copolymer structure; thus, not all the acrylic functionalities were active during the polymerization process under the experimental conditions. The presence of ASO provides flexibility to the copolymers, and it decreases the glass transition temperature of the neat acrylic copolymer (from −7.2 °C to −22.8 °C). Hydrophobic and oleophobic properties are also controlled by the presence of ASO on the copolymers and by the polymer composition.

## Figures and Tables

**Table 1:** Miniemulsion formulations based on 100 g of monomer.

Reference	MMA (g)	BA (g)	AA (g)	SA (g)	Biomonomer (g)	SDS (g)	H_2_O (g)
B_0	45	54	1	4	-	2	150
B_ASO5_20	25	54	1	4	20 ASO5	2	150
B_ASO8_20	25	54	1	4	20 ASO8	2	150
B_ASO11_20	25	54	1	4	20 ASO11	2	150

**Table 2:** ASO systems selected for the study.

Reference	MR Cat/C=C	MR AA/C=C	Acrylation Degree	Process
ASO5	0.1	6	2.5	Batch
ASO8	0.1	4	1.6	Batch
ASO11	0.1	4	1.9	Semicontinuous

**Table 3:** Different polymerization data.

Reference	dd (nm)	dp (nm)	Nd	Np	Np/Nd
B0	87	114	2.3 × 10^17^	1.0 × 10^17^	0.43
B_ASO5_20	110	132	1.1 × 10^17^	6.3 × 10^16^	0.6
B_ASO8_20	107	108	1.2 × 10^17^	1.2 × 10^17^	1
B_ASO11_20	110	108	1.1 × 10^17^	1.3 × 10^17^	1.1

## Data Availability

The data presented in this study are available upon request from the corresponding authors.

## Supplementary Materials

The following supporting information can be downloaded at https://www.mdpi.com/article/10.3390/polym16162355/s1. Figure S1: Assignation of ^1^H NMR peaks of SO molecule; Figure S2: Assignation of ^1^H NMR peaks of ASO molecule; Figure S3: FTIR spectrum of ASO biomonomers (ASO5, ASO8 and ASO11). Equation (S1): Determination of the number of C=C in SO; and Equation (S2): Acrylation degree quantification of ASO.
