# Supplementary material for: Direct Acrylation of Soybean Oil and the Influence of the Acrylation Degree on Waterborne Acrylic Systems
Source: Polymers (Basel). 2024 Aug 20;16(16):2355. doi: 10.3390/polym16162355 (PMC11358965; doi:10.3390/polym16162355)
Supplement: Supplementary file 1 [file polymers-16-02355-s001.zip › polymers-3120303-supplementary.pdf]

Determination of the number of the C=C in SO

The determination of the number of the C=C present in SO was carried out by comparing the peak area of Hh (2.3 ppm) to that of Hd (5.5 ppm) (Figure S1). The peak area of Hd was determined by subtraction of the proton of He (due to the glycerol residue) from the total peak area of Hd plus He. The average number of double bonds in SO was determined using the Equation[11]

$$C = C \text{ in SO} = \frac{\text{Ad+e}-\text{Ag}/4\,/_{2}}{\text{Ai}/6} \qquad \text{(Equation S1)}$$

Acrylation degree quantification

The peaks at 4.1–4.4 ppm is due to the protons in the methylene groups of the triglyceride (Figure S2). The vinylic hydrogens are detected at 5.2–5.5 ppm. The protons in the CH_2 groups between two carbon–carbon double bonds appear at 2.0–2.5 ppm. The methylene and methyl protons of acrylate end group showed signal at 6.34 and 5.87 ppm. The peak areas of the protons associated with acrylate groups (Ha, Hb, Hc, and Hf) increased with the rise in acrylation degree, while the peak areas of the protons associated with double bonds (Hd, Hh, and Hj) decreased in the same way.

To quantify the acrylates groups/TG the signals around 4.25 ppm reference the 4 protons of the methylene groups that originally come from glycerol were integrated. Then, taking these values as a reference, the 3 signals between 5.7-6.5 ppm quantify the 3 expected protons per each introduced acrylic molecule were also integrated. The acrylic groups per molecule, were calculated as follows:

$$\frac{\text{Integral acrylate peak}}{\text{Integral reference}}\,\frac{4}{3} \qquad \text{(Equation S2)}$$

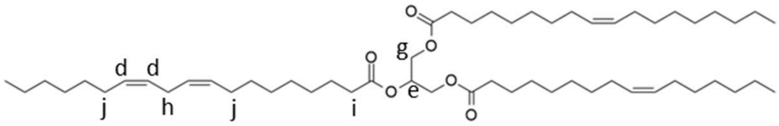

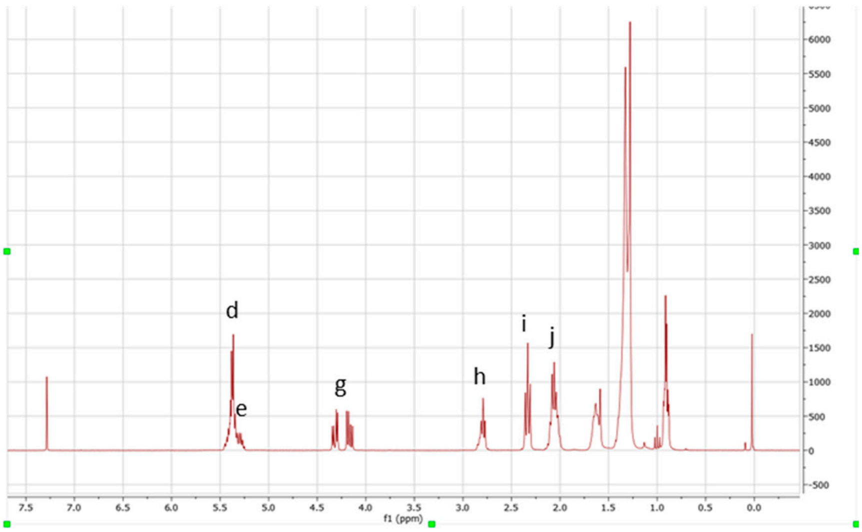

Figure S1 Assignation of H+NMR peaks of SO molecule.

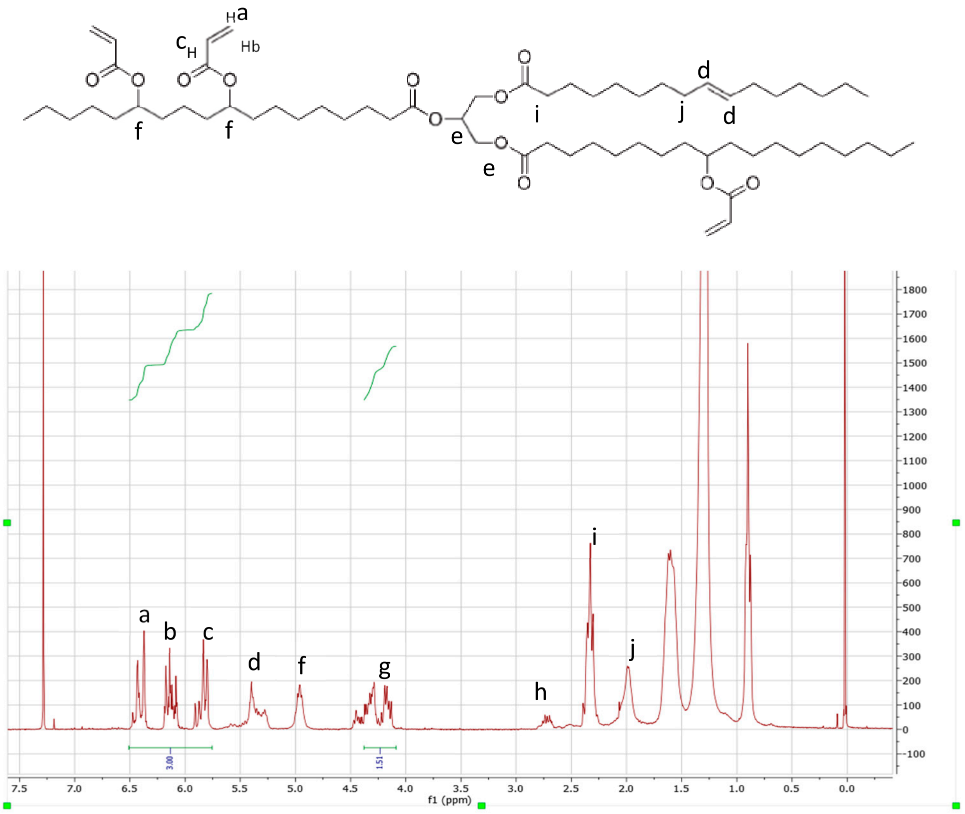

Figure S2 Assignation of H+NMR peaks of ASO molecule.

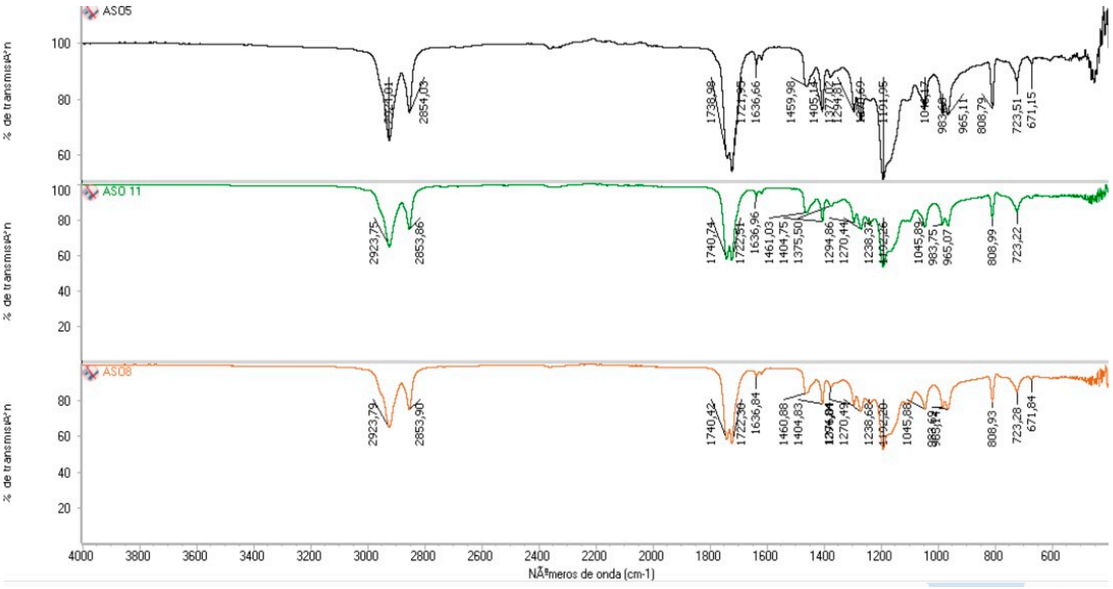

Figure S3 FTIR spectrum of ASO biomonomers (ASO5, ASO8 and ASO11).
